# Supplementary material for: Randomized resonant metamaterials for single-sensor identification of elastic vibrations
Source: Nat Commun. 2020 May 11;11:2353. doi: 10.1038/s41467-020-15950-1 (PMC7214442; doi:10.1038/s41467-020-15950-1)
Supplement: Supplementary file 1 — Supplementary Information [file 41467_2020_15950_MOESM1_ESM.pdf]

Supplementary Information

Randomized resonant metamaterials for single-sensor identification of elastic vibrations

Jiang *et al*.

Supplementary Information includes:

Supplementary Figure 1. Effective mass of a local resonator with the resonant frequency of 541 Hz.
Supplementary Figure 2. Node configuration of the local resonator in numerical simulations.
Supplementary Figure 3. Node configuration and boundary conditions of the randomly coupled resonator system.
Supplementary Figure 4. Transmission property of the entire coupling system.
Supplementary Figure 5. The relationship between the μ_{Ave} and the parameters of the randomly coupled resonator system.
Supplementary Figure 6. Effects of the number of the local resonators on the μ_{Ave}.
Supplementary Figure 7. Waveforms and spectra of the twenty testing signals in the signal set.
Supplementary Figure 8. An example of the signal processing details for vibration identification.
Supplementary Figure 9. Entire reconstruction results of 40 experiments for Config. 1.
Supplementary Figure 10. Simplified reconstruction results of 40 experiments for Config. 1.
Supplementary Figure 11. Reconstruction results of Config. 2 with two or three activated sources.
Supplementary Figure 12. Reconstruction results of Config. 3.
Supplementary Figure 13. Details of the vibration identification for trajectory tracking.
Supplementary Figure 14. Tracking process of the trajectories "SJTU".
Supplementary Figure 15. Tracking process of the trajectory "Vase".

Supplementary Table 1. Parameters of the randomly coupled resonator system.
Supplementary Table 2. Node connection of the coupling network.
Supplementary Table 3. Parameters of the unit cells in six supercells.

Supplementary Note 1. Evaluation of the uncorrelation of vibration transmissions.
Supplementary Note 2. Parameter effects on the vibration transmission property of the randomly coupled resonator system.
Supplementary Note 3. Details of signal processing for multi-source vibration identification.
Supplementary Note 4. Construction of the measurement matrix for impact identification.
Supplementary Note 5. Details of the vibration identification for trajectory tracking.

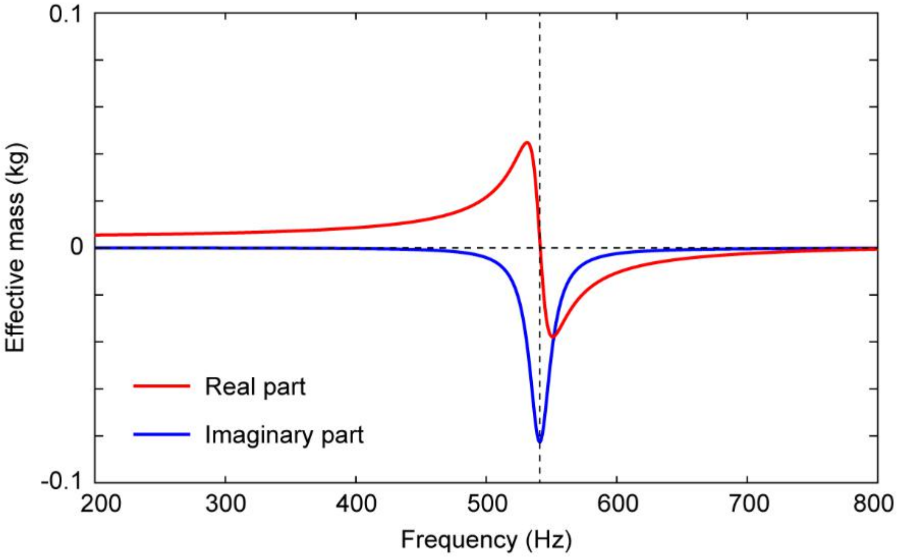

Supplementary Figure 1 | Effective mass of a local resonator with the resonant frequency of 541 Hz. It can be seen that the real part and imaginary part of the effective mass are negative near the resonant frequency, which means that the vibration decays exponentially as it propagates.

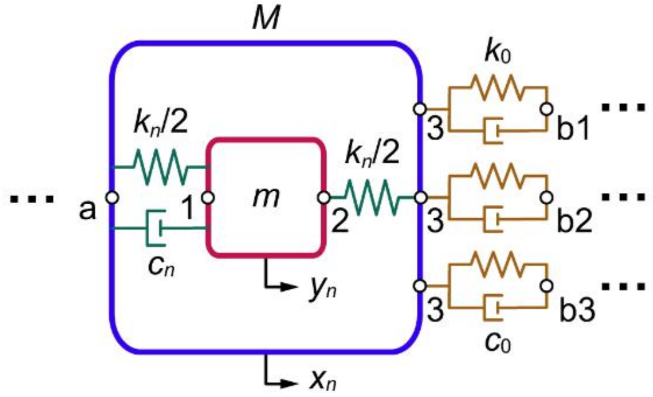

Supplementary Figure 2 | Node configuration of the local resonator in numerical simulations.

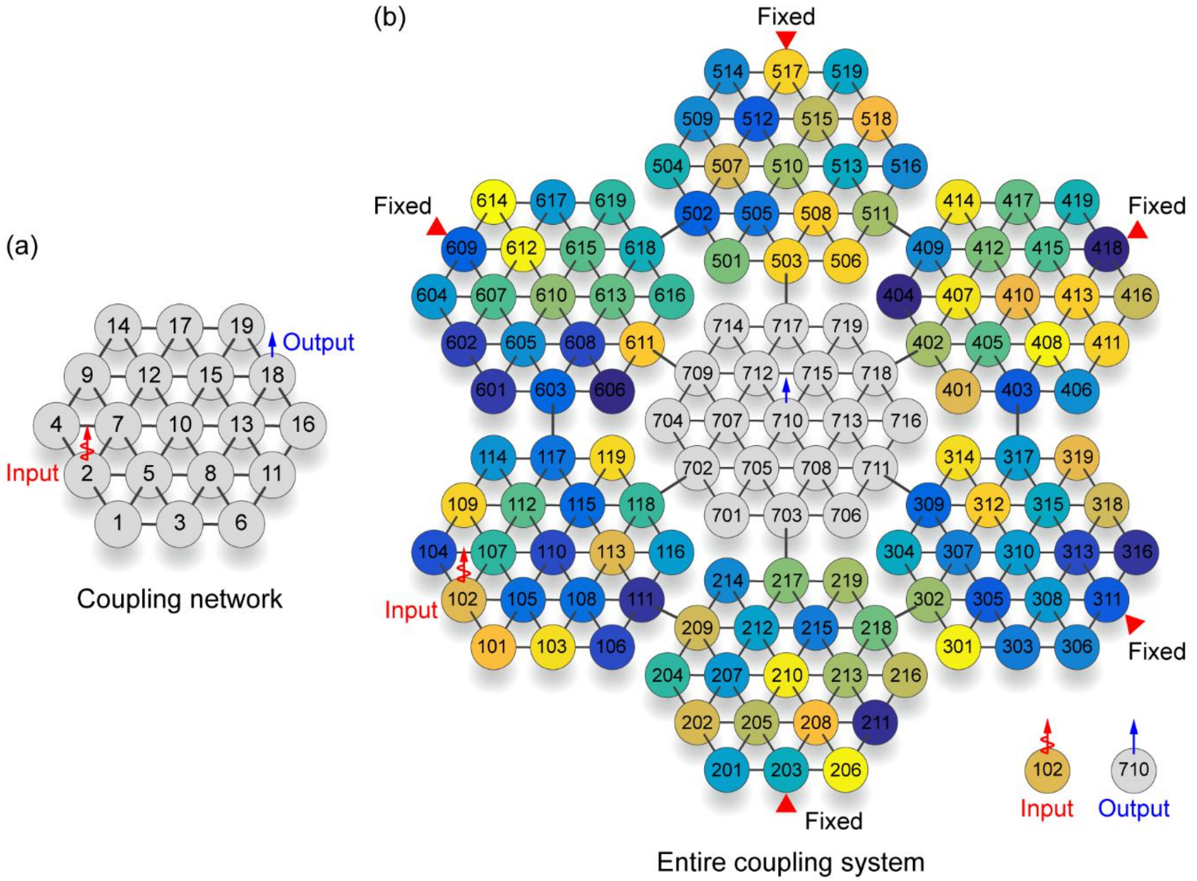

Supplementary Figure 3 | Node configuration and boundary conditions of the randomly coupled resonator system. (a) Node connection of the coupling network. (b) Node configuration and boundary conditions of the entire coupling system.

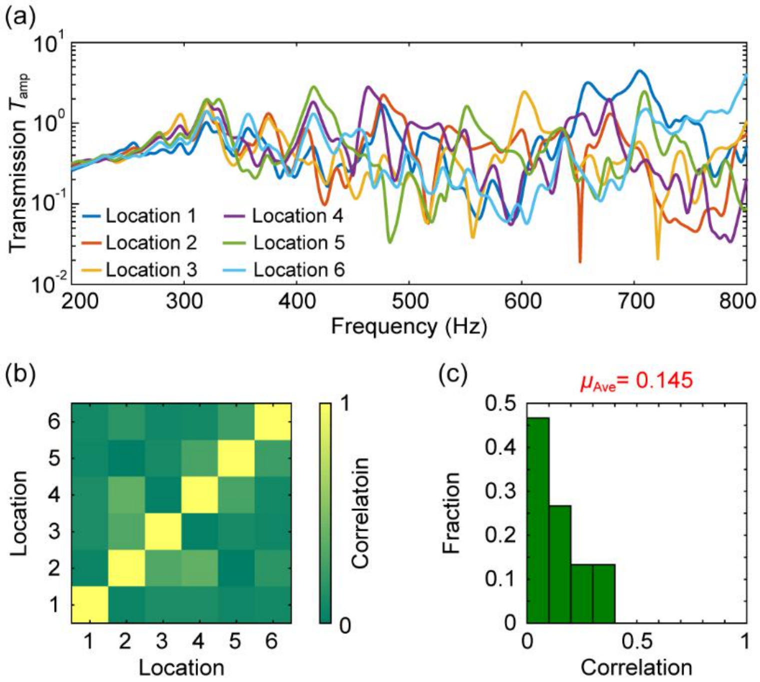

Supplementary Figure 4 | Transmission property of the entire coupling system. (a) Transmissions of the entire coupling system for six different locations. (b) Absolute correlation coefficients of the transmissions. (c) Histogram of the absolute cross-correlation coefficients.

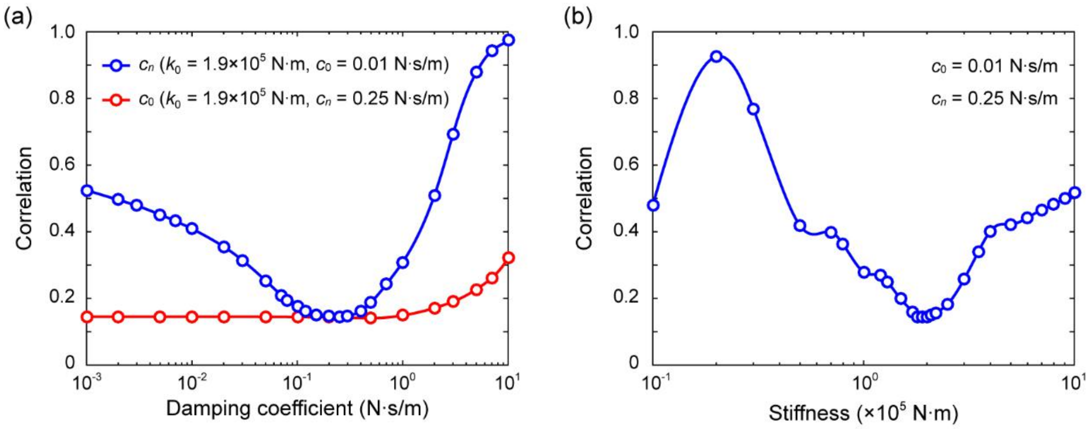

Supplementary Figure 5 | The relationship between the μ_{Ave} and the parameters of the randomly coupled resonator system. (a) Effects of the damping c_n and c_0 when $k_0 = 1.9 \times 10^5$ N m^{-1}. (b) Effects of the matrix stiffness k_0 when $c_0 = 0.01$ N s m^{-1} and $c_n = 0.25$ N s m^{-1}.

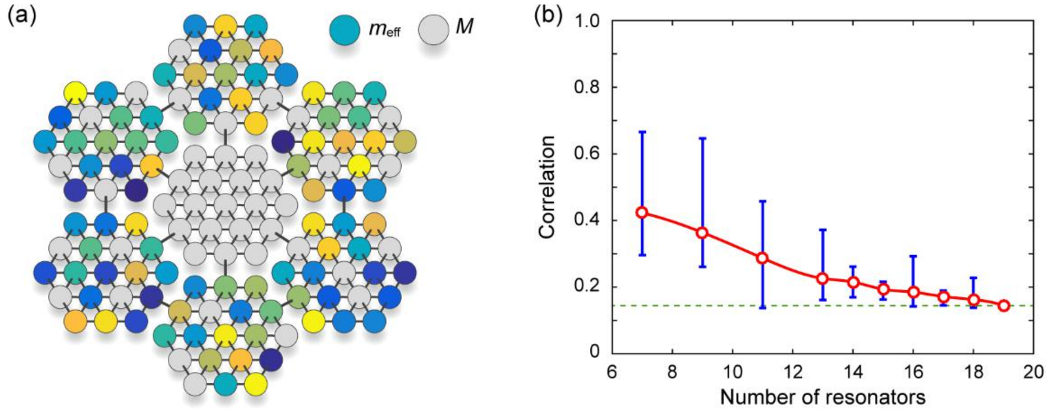

Supplementary Figure 6 | Effects of the number of the local resonators on the μ_{Ave}. (a) Schematic of randomly replacing four effective masses m_n^{eff} with four mass M in each coupling network. (b) The relationship between the μ_{Ave} and the number of the local resonators in each coupling network. Here, each point denotes the average of the μ_{Ave} in 10 calculations, and the error bar denotes the maximum and minimum values of the μ_{Ave} in 10 calculations.

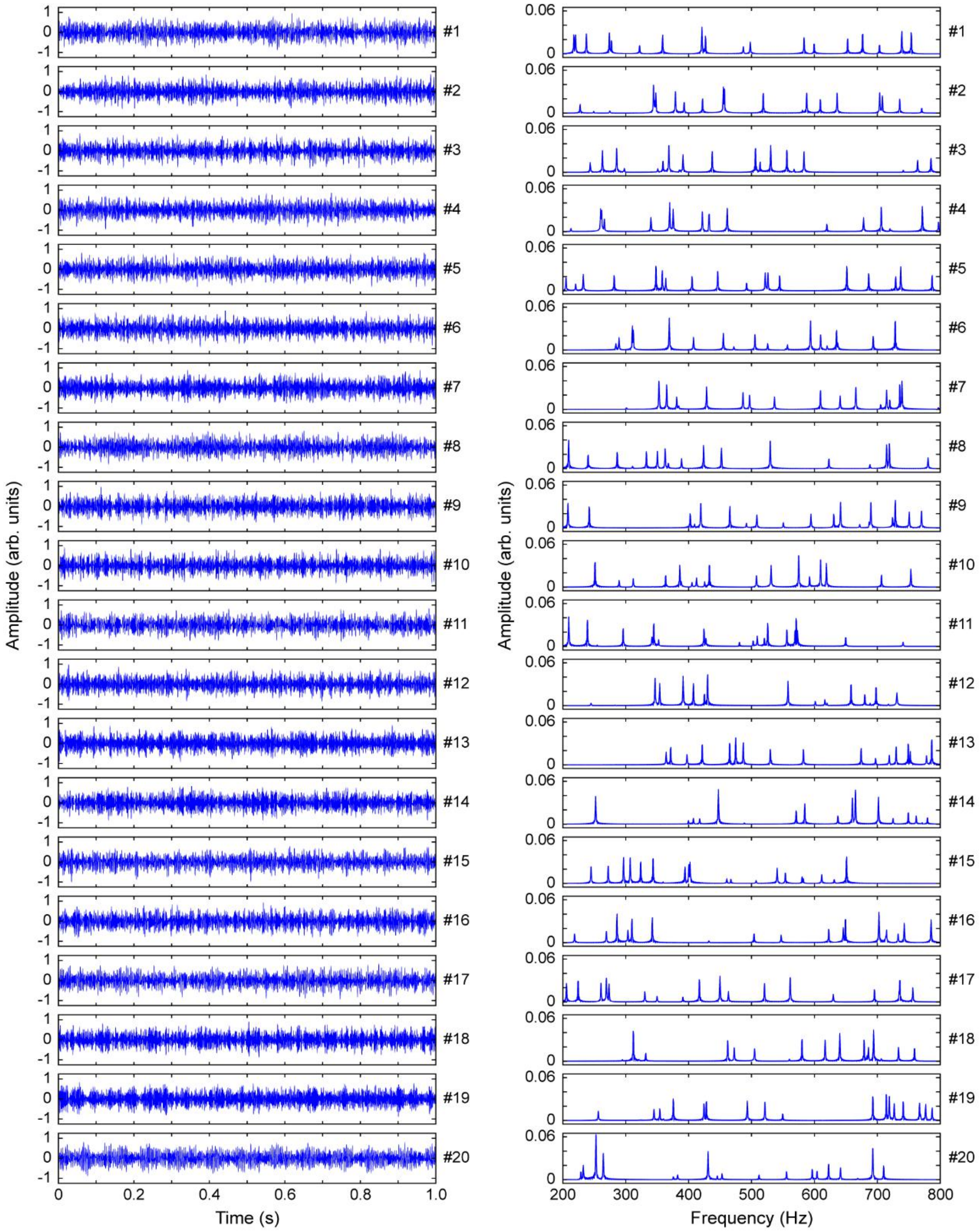

Supplementary Figure 7 | Waveforms and spectra of the 20 testing signals in the signal set. The testing signals are broadband random signals.

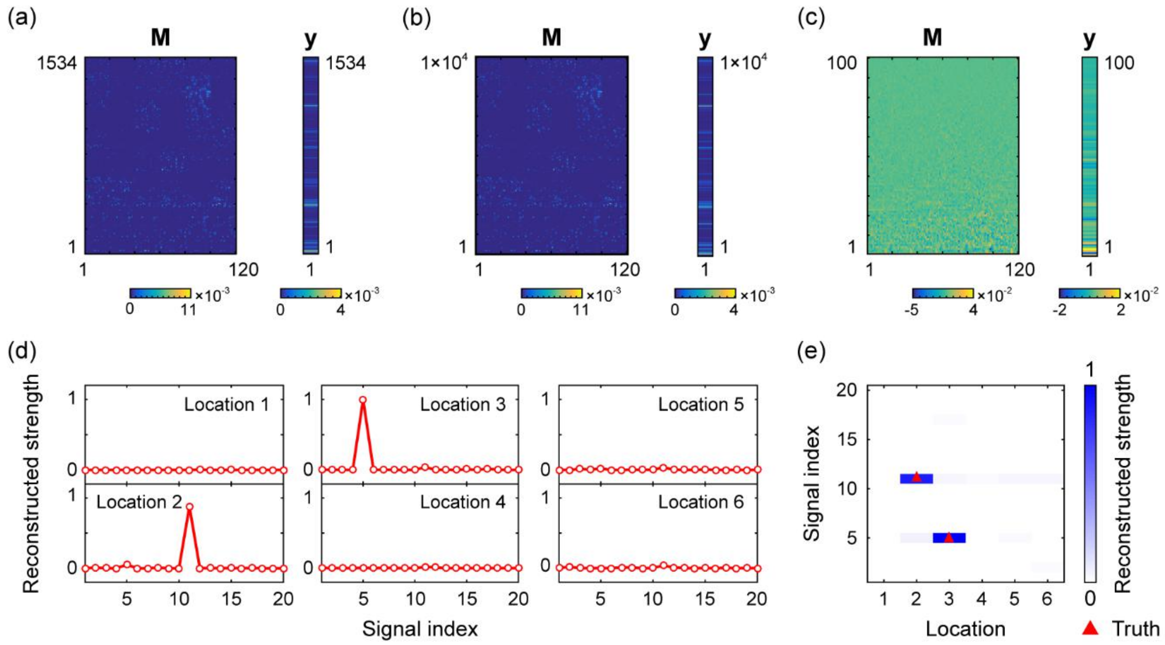

Supplementary Figure 8 | An example of the signal processing details for vibration identification. (a) The calibrated measurement matrix **M** and observed vector **y**. (b) The replicated **M** and **y**. (c) The compressed **M** and **y** by using principal component analysis. (d) The details and (e) the visualization of the reconstructed results.

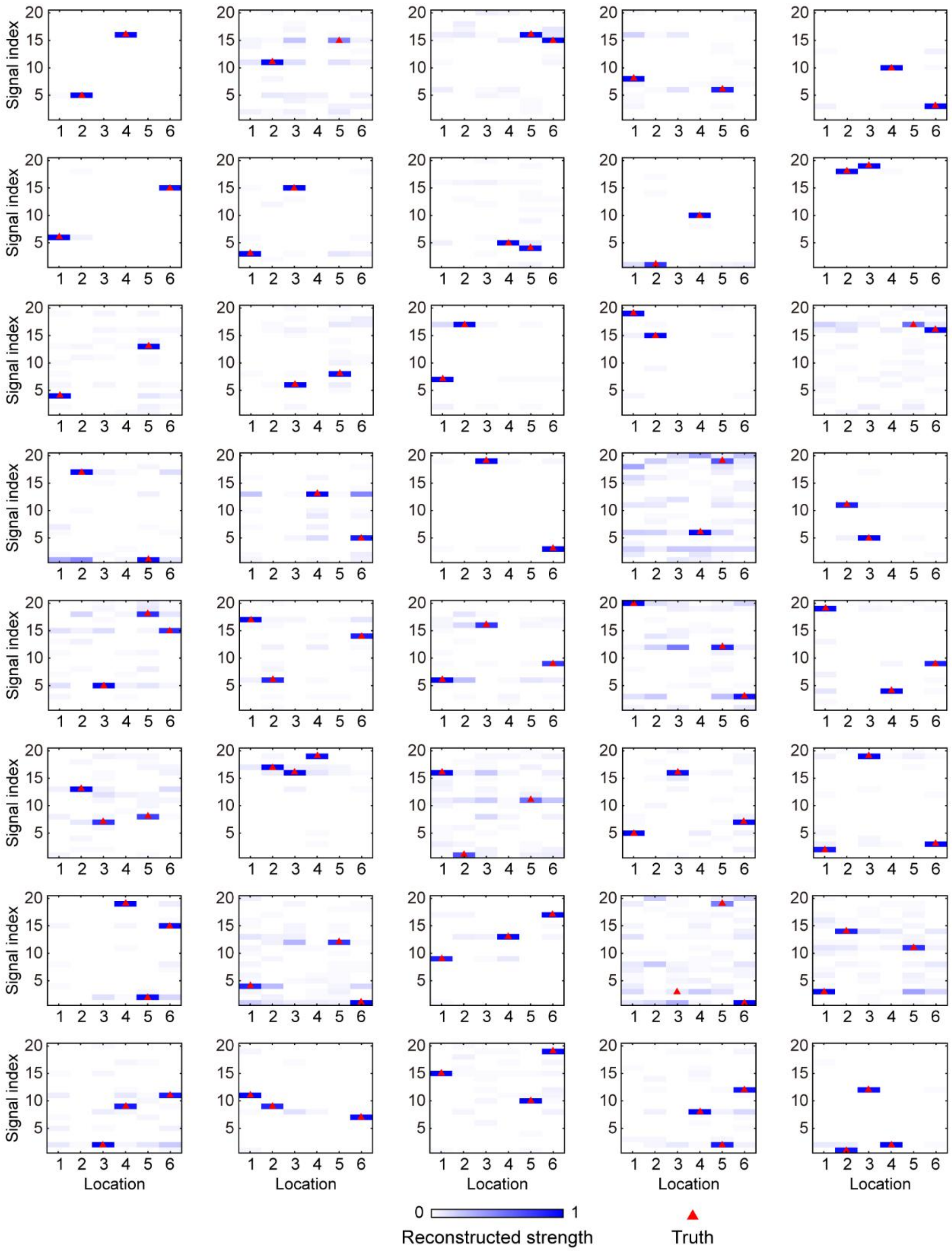

Supplementary Figure 9 | Entire reconstruction results of 40 experiments for Config. 1.

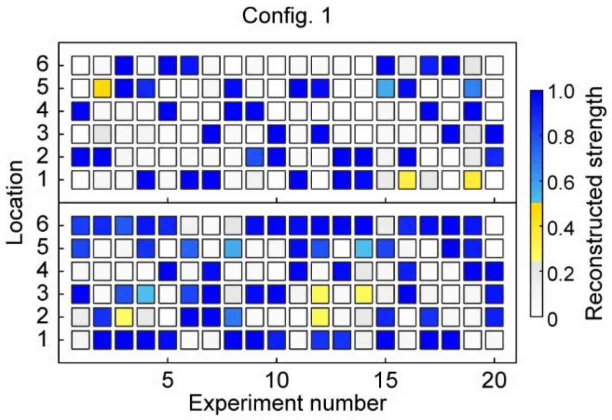

Supplementary Figure 10 | Simplified reconstruction results of 40 experiments for Config. 1. The locations of the vibration sources can be clearly reflected in the images if they are correctly identified. The recognition ratio of the Config. 1 is 96.7%.

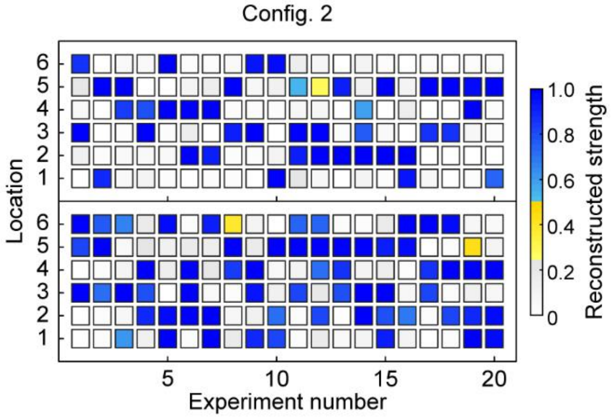

Supplementary Figure 11 | Reconstruction results of Config. 2 with two or three activated sources. The recognition ratio of Config. 2 is 97.9%.

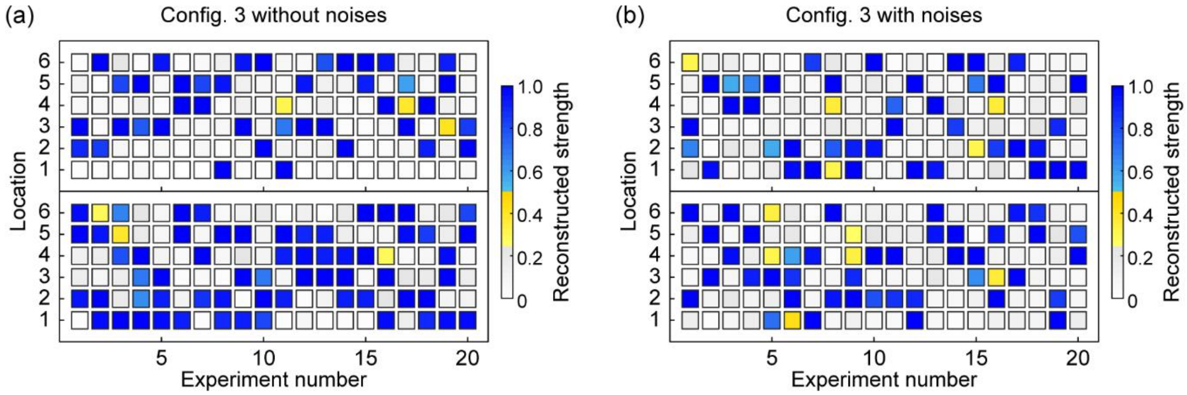

Supplementary Figure 12 | Reconstruction results of Config. 3. (a) The reconstruction results with two or three activated sources. The recognition ratio is 97.1%. (b) The reconstruction results of Config. 3 with interferences of Gaussian noises. The recognition ratio is 95.0%.

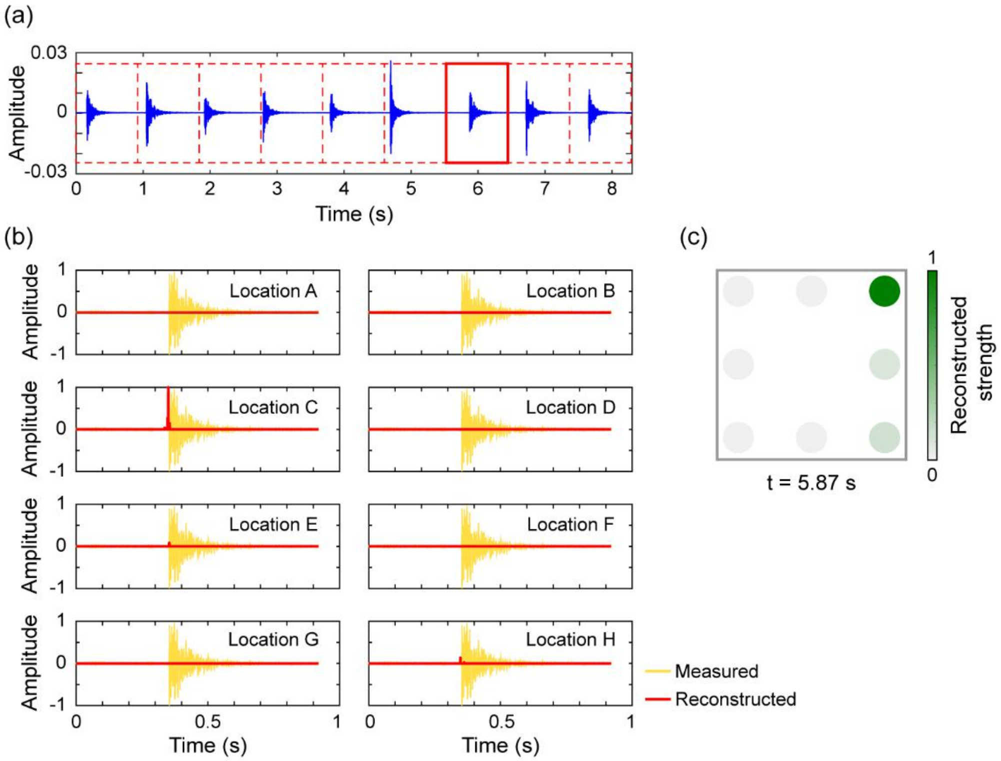

Supplementary Figure 13 | Details of the vibration identification for trajectory tracking. (a) The measured signal from the single sensor. (b) The normalized reconstruction results of the 7th vibration event, and (c) the visualization of the maximum values in the reconstructed vector segments.

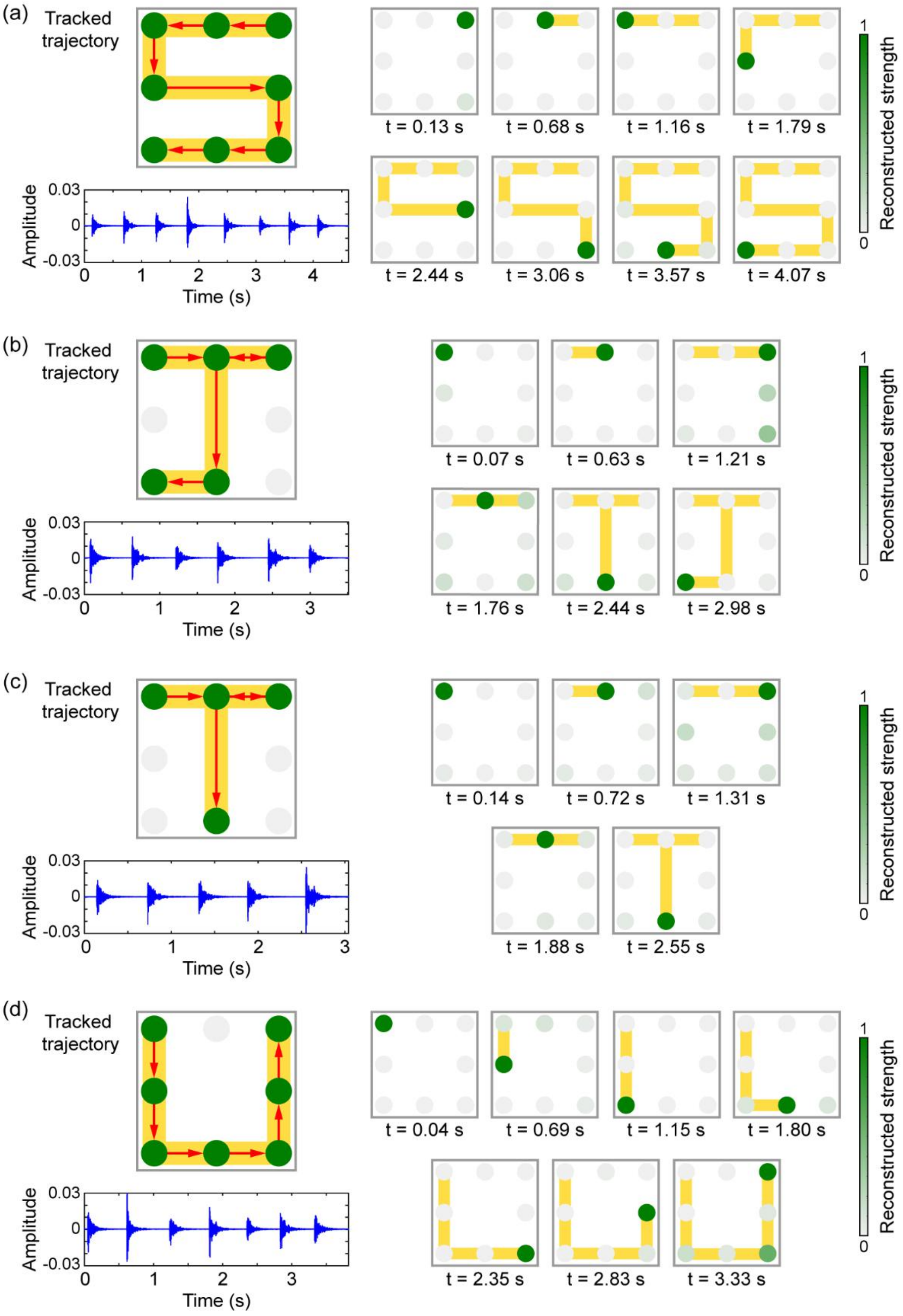

Supplementary Figure 14 | Tracking process of the trajectories "SJTU". (a)-(d) The tracked trajectories, measured signals, and reconstruction results of "S", "J", "T", and "U", respectively.

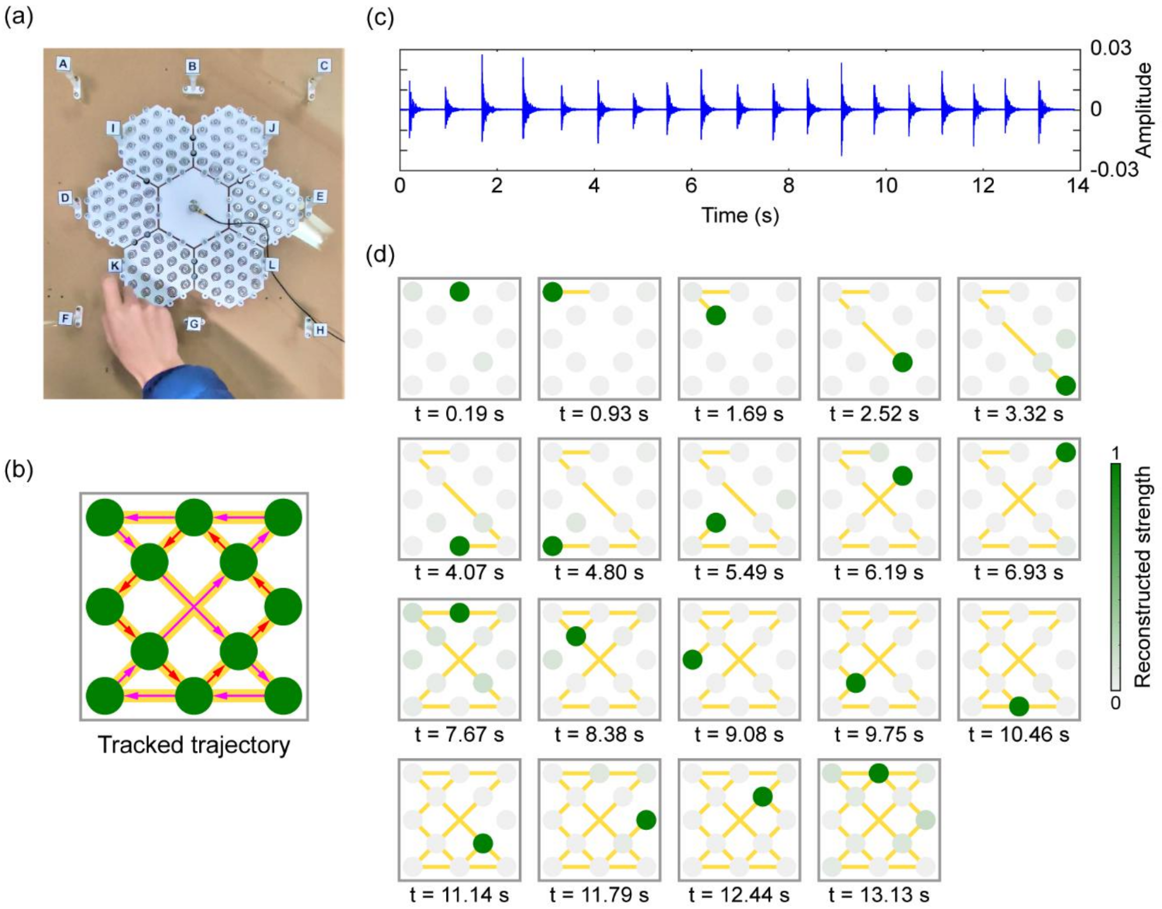

Supplementary Figure 15 | Tracking process of the trajectory "Vase". (a) Experimental setup. (b) The tracked trajectory. (c) The measured signal from the single sensor. (d) Reconstruction results of each vibration event.

Supplementary Table 1 | Parameters of the randomly coupled resonator system.

Local resonator	Coupling network #1		Coupling network #2		Coupling network #3		Coupling network #4		Coupling network #5		Coupling network #6	
	k_n (kN/m)	f_n (Hz)	k_n (kN/m)	f_n (Hz)	k_n (kN/m)	f_n (Hz)	k_n (kN/m)	f_n (Hz)	k_n (kN/m)	f_n (Hz)	k_n (kN/m)	f_n (Hz)
1	84.90	846.68	21.35	424.57	113.12	977.32	76.23	802.27	48.36	638.98	2.82	154.42
2	73.96	790.23	72.21	780.86	55.77	686.21	58.31	701.66	6.48	233.99	3.66	175.82
3	104.88	941.03	26.44	472.51	11.24	308.11	5.74	220.15	98.02	909.74	7.26	247.51
4	4.60	197.10	34.93	543.11	25.40	463.14	1.69	119.40	30.25	505.35	18.18	391.80
5	8.25	264.00	62.30	725.27	5.21	209.82	43.19	603.86	9.63	285.11	16.35	371.55
6	4.24	189.19	112.69	975.46	13.82	341.59	16.28	370.74	98.01	909.69	1.45	110.51
7	34.64	540.79	18.48	394.98	13.06	332.06	105.87	945.47	73.23	786.33	40.66	585.91
8	8.89	273.92	86.38	854.02	18.81	398.50	114.40	982.81	94.71	894.24	4.09	185.84
9	97.28	906.30	69.34	765.17	6.65	237.01	15.18	357.96	15.05	356.46	6.37	231.86
10	4.24	189.18	108.87	958.76	20.22	413.21	79.78	820.74	59.02	705.90	52.85	668.03
11	2.31	139.75	1.96	128.73	5.20	209.49	97.32	906.50	57.68	697.85	90.34	873.39
12	42.86	601.57	21.01	421.18	95.03	895.74	48.17	637.77	5.25	210.53	113.00	976.80
13	74.90	795.25	57.44	696.39	4.05	184.85	95.00	895.62	25.78	466.59	44.61	613.75
14	17.17	380.75	14.79	353.35	103.99	937.04	106.74	949.36	14.32	347.76	117.76	997.17
15	8.07	261.08	11.19	307.34	24.96	459.12	41.82	594.24	65.74	745.00	42.38	598.19
16	19.43	405.06	64.86	740.02	2.41	142.66	67.61	755.55	14.93	355.05	37.66	563.91
17	9.90	289.13	51.92	662.12	19.73	408.14	45.39	619.08	97.34	906.58	18.72	397.61
18	37.03	559.14	47.24	631.55	68.84	762.37	1.80	123.27	84.35	843.92	28.09	487.00
19	99.31	915.73	57.11	694.39	78.71	815.21	29.83	501.88	24.09	451.02	34.87	542.63

Supplementary Table 2 | Node connection of the coupling network.

Node	Local resonator									
	#1	#2	#3	#4	#5	#6	#7	#8	#9	#10
a	Free	2, Input	3	4	5	6	7	8	9	10
b1	2	4	5	Free	7	8	9	10	Free	12
b2	5	7	8	9	10	11	12	13	14	15
b3	3	5	6	7	8	Free	10	11	12	13

Node	Local resonator								
	#11	#12	#13	#14	#15	#16	#17	#18	#19
a	11	12	13	14	15	16	17	18, Output	19
b1	13	14	15	Free	17	18	Free	19	Free
b2	16	17	18	Free	19	Free	Free	Free	Free
b3	Free	15	16	17	18	Free	19	Free	Free

Supplementary Table 3 | Parameters of the unit cells in six supercells.

Unit cell	Supercell #1		Supercell #2		Supercell #3		Supercell #4		Supercell #5		Supercell #6	
	θ_n (rad)	f_n (Hz)	θ_n (rad)	f_n (Hz)	θ_n (rad)	f_n (Hz)	θ_n (rad)	f_n (Hz)	θ_n (rad)	f_n (Hz)	θ_n (rad)	f_n (Hz)
1	10.672	121.16	10.654	122.26	8.699	490.52	11.417	89.79	8.667	504.83	9.069	355.28
2	9.041	363.75	8.517	578.94	9.659	223.61	9.958	181.94	9.637	227.10	8.854	427.23
3	9.243	307.59	8.502	587.18	9.107	344.05	11.801	80.13	9.941	183.98	9.505	250.52
4	14.111	54.65	9.029	367.38	11.121	99.75	9.308	292.07	9.579	236.96	9.972	180.20
5	9.597	233.87	11.655	83.44	10.854	111.41	13.948	55.67	9.481	255.24	9.022	369.49
6	10.413	138.48	9.994	177.75	10.616	124.58	8.567	552.77	9.083	351.12	8.603	535.13
7	8.693	493.02	9.547	242.74	8.856	426.42	10.192	157.07	9.054	359.83	9.396	272.54
8	9.684	219.58	8.659	508.60	9.187	322.10	11.075	101.58	9.425	266.44	10.844	111.89
9	9.118	340.92	13.721	57.18	10.233	153.32	9.697	217.46	9.418	267.72	9.303	293.26
10	10.388	140.35	12.117	74.15	9.187	322.00	9.274	300.11	9.145	333.49	9.723	213.51
11	8.982	382.60	10.365	142.14	10.522	130.65	10.969	106.03	8.889	414.32	9.410	269.54
12	9.868	193.12	8.903	409.50	11.770	80.81	8.461	610.17	8.508	584.13	8.644	515.66
13	8.895	412.34	8.778	456.91	8.622	525.73	9.613	231.09	8.792	451.51	9.404	270.79
14	8.840	432.47	8.905	408.72	9.056	359.06	9.733	211.96	9.413	268.81	9.440	263.29
15	8.755	466.37	9.286	297.23	8.531	571.68	11.605	84.68	8.645	514.79	9.525	246.78
16	9.287	297.01	9.082	351.38	8.829	436.75	9.730	212.45	10.633	123.53	10.582	126.70
17	11.211	96.46	9.737	211.39	9.015	371.69	12.010	76.02	11.639	83.82	9.880	191.57
18	10.021	174.59	8.760	464.38	8.666	505.24	9.165	327.88	11.204	96.69	11.166	98.07
19	8.549	562.18	10.238	152.91	8.588	542.18	8.737	474.03	10.400	139.40	9.338	285.17

Supplementary Note 1. Evaluation of the uncorrelation of vibration transmissions

The vibration transmissions of the metamaterial are contributed to the construction of the measurement matrix, which directly influences the performance of the vibration identification. Supplementary Figure 4(a) shows the transmissions of the coupling system. The correlation coefficients μ_{ij} of the transmissions is calculated as

$$\mu_{ij} = \frac{\sum_p \left[H_i(\omega_p) - \bar{H}_i\right]\left[H_j(\omega_p) - \bar{H}_j\right]}{\sqrt{\sum_p \left(H_i(\omega_p) - \bar{H}_i\right)^2}\sqrt{\sum_p \left(H_j(\omega_p) - \bar{H}_j\right)^2}}, \tag{1}$$

where p is the index of the frequency, $H_i(\omega_p)$ and $H_j(\omega_p)$ are transmissions of the coupling system, $\bar{H}_i$ and $\bar{H}_j$ are the averages of $H_i(\omega_p)$ and $H_j(\omega_p)$. Here, we define the correlation matrix $\mathbf{C}_\mu$ as

$$\mathbf{C}_\mu = \begin{bmatrix} \mu_{11} & \cdots & \mu_{1k} \\ \vdots & \ddots & \vdots \\ \mu_{k1} & \cdots & \mu_{kk} \end{bmatrix}, \tag{2}$$

where k is the number of source locations. Supplementary Figure 4(b) visualizes the absolute values of the elements in $\mathbf{C}_\mu$ (i.e. $|\mu_{ij}|$) for the coupling system. To quantify the uncorrelation of the vibration transmissions, the average of the absolute cross-correlation coefficients μ_{Ave} is defined as

$$\mu_{\text{Ave}} = \frac{1}{k(k-1)} \sum_{i \neq j} \left|\mu_{ij}\right|. \tag{3}$$

Here, μ_{Ave} ranges from an ideal 0 (i.e. perfectly orthogonal modulation) to a useless 1 (i.e. identical modulation). To avoid the existence of extreme values close to 1 (while μ_{Ave} is still small), we plot the histogram to show the distribution of the absolute cross-correlation coefficients in Supplementary Figure 4(c). In this way, we can make sure that the correlations of each two transmissions are small. In the histogram, the absolute cross-correlation coefficients distribute narrowly around zero, indicating that the transmissions are highly uncorrelated.

Supplementary Note 2. Parameter effects on the vibration transmission property of the randomly coupled resonator system

The absolute average cross-correlation μ_{Ave} is a metric to evaluate the uncorrelation of the vibration transmission, which is highly related to the identification performance. To study the parameter effects on the transmission property of the randomly coupled resonator system, we calculated the μ_{Ave} under different c_n (damping of the resonator), c_0 (damping of the matrix) and k_0 (stiffness of the matrix). The results are shown in Supplementary Figure 5. It can be seen that c_n and k_0 have a great influence on μ_{Ave}, while c_0 has a relatively small effect on μ_{Ave}. Therefore, materials with appropriate damping and stiffness should be selected to achieve the optimum identification performance.

The coupling network contains 19 local resonators. To study the effect of reducing the number of the local resonators on the vibration transmission property, we randomly replace the effective masses m_n^{eff} with the matrix masses M in each coupling network, and calculate the μ_{Ave} of the entire system (Supplementary Figure 6(a)). Each random replacement process is conducted 10 times, and the average, maximum, minimum values of the μ_{Ave} are obtained from 10 times of calculations as shown in Supplementary Figure 6(b). It can be seen that the average of the μ_{Ave} is enlarged by reducing the number of local resonators, but the μ_{Ave} in some special cases is smaller than that with 19 resonators. This means that by further optimizing the design, the maximum uncorrelation of the transmissions can be achieved with minimum quantities of resonators.

Supplementary Note 3. Details of signal processing for multi-source vibration identification

In our experiments, the measurement matrix $\mathbf{M}_{p\times q}$ is experimentally calibrated by successively playing the 20 testing signals from six different locations and calculating the spectra of the measured signals (see Supplementary Figure 8(a)). The operational frequency range is 200 ~ 800 Hz, the sampling frequency is 12.82 kHz, p = 1534, and q = 120. The observation vector $\mathbf{y}_{p\times 1}$ is obtained by playing testing signals from multiple sources at the same time. Then, we replicates the matrices $\mathbf{y}_{p\times 1}$ and $\mathbf{M}_{p\times q}$ by row (now, $p = 1 \times 10^4$) to increase the weight of the effective information as shown in Supplementary Figure 8(b). Next, principal component analysis is used to compress the dimensionality of the measurement matrix. Here, the first 100 principal singular values are selected. The compressed **M** and **y** are presented in Supplementary Figure 8(c).

The solution of $\mathbf{y} = \mathbf{Mx}$ can be obtained with an L1-norm minimization

$$\hat{\mathbf{x}} = \min \|\mathbf{x}\|_1 , \quad \mathrm{s.t.}\ \mathbf{Mx} = \mathbf{y} , \tag{4}$$

where $\hat{\mathbf{x}}$ is the estimation of the unknown **x**, and $\|\mathbf{x}\|_1$ denotes the L1-norm $\sum_i |\mathbf{Mx}_i - \mathbf{y}_i|$ of **x**. We use the two-step iterative shrinkage/thresholding algorithm to solve this inverse problem. The normalized absolute $\hat{\mathbf{x}}$ can be divided into six parts according to the number of locations as shown in Supplementary Figure 8(d). It can be seen that the testing signal #11 is generated from Location 2, and the testing signal #5 is generated from Location 3, which agrees well with the truth. Finally, we visualize and present the reconstructed result in Supplementary Figure 8(e).

Supplementary Note 4. Construction of the measurement matrix for impact identification

The convolution process of impact identification for the randomized resonant metamaterial system can be expressed as

$$y(t) = \int_0^t h(t-\tau) f(\tau)\, \mathrm{d}\tau , \tag{5}$$

where $y(t)$ is the observation signal, $h(t)$ is the impulse response function, and $f(t)$ is the impact function. The discrete form of Supplementary Equation 5 can be given by

$$y(n\Delta t) = \Delta t \sum_{i=1}^{n} \left[h\left((n-i+1)\Delta t\right) \cdot f(i\Delta t) \right], \tag{6}$$

where Δt is the time interval. Supplementary Equation 6 can be written as the following matrix-vector form

$$\begin{bmatrix} y(\Delta t) \\ y(2\Delta t) \\ \vdots \\ y((n-1)\Delta t) \\ y(n\Delta t) \end{bmatrix} = \Delta t \begin{bmatrix} h(\Delta t) & 0 & \cdots & 0 & 0 \\ h(2\Delta t) & h(\Delta t) & \cdots & 0 & 0 \\ \vdots & \vdots & \ddots & \vdots & \vdots \\ h((n-1)\Delta t) & h((n-2)\Delta t) & \cdots & h(\Delta t) & 0 \\ h(n\Delta t) & h((n-1)\Delta t) & \cdots & h(2\Delta t) & h(\Delta t) \end{bmatrix} \begin{bmatrix} f(\Delta t) \\ f(2\Delta t) \\ \vdots \\ f((n-1)\Delta t) \\ f(n\Delta t) \end{bmatrix}. \tag{7}$$

Therefore, the measurement matrix for impact identification can be constructed by continuously delaying the directional impulse responses of the metamaterial system with time interval Δt in a time window. In our experiments, the impulse responses of the metamaterial system are directly obtained by applying impulses to different locations. Then, time alignment and amplitude normalization are performed to obtain the calibrated impulse responses $h^k(t)$, where k is the location index. The measurement matrix $\mathbf{M}$ for impact identification can be constructed as $\mathbf{M} = [\mathbf{H}^1, \mathbf{H}^2, \ldots, \mathbf{H}^6]$, where

$$\mathbf{H}^k = \begin{bmatrix} h^k(\Delta t) & 0 & \cdots & 0 & 0 \\ h^k(2\Delta t) & h^k(\Delta t) & \cdots & 0 & 0 \\ \vdots & \vdots & \ddots & \vdots & \vdots \\ h^k((n-1)\Delta t) & h^k((n-2)\Delta t) & \cdots & h^k(\Delta t) & 0 \\ h^k(n\Delta t) & h^k((n-1)\Delta t) & \cdots & h^k(2\Delta t) & h^k(\Delta t) \end{bmatrix}, \quad k = 1, 2, \ldots, 6. \tag{8}$$

Supplementary Note 5. Details of the vibration identification for trajectory tracking

The waveform presented in Fig. 5(a) is the whole measured signal in the trajectory tracking. We continuously intercept the measured signal according to each vibration event as shown in Supplementary Figure 13(a). Here we take an example to show the identification performance, where the 7th fragment of the measured signal is used to be the observation vector. Supplementary Figure 13(b) shows the normalized reconstruction result of the selected vibration event. The occurrence location and time of the reconstructed impact are in good agreement with the actual one. We calculate the maximum value of the reconstructed vector segment corresponding to each location in Supplementary Figure 13(b). The maximum values are visualized in Supplementary Figure 13(c). It can be seen that an impact is applied to the probe C. Furthermore, we use a similar method to reconstruct the trajectories “SJTU” as shown in Supplementary Figure 14.

Supplementary Figure 15 shows the reconstruction details of the trajectory “Vase”, where the number of probes is increased to 12. It can be seen that the occurrence locations and time of the vibration events can still be successfully identified. This tracking process is dynamically shown in Supplementary Movie 2. The results above demonstrate that the proposed device can be used to track complex trajectories, which has potential application prospects in fields such as human-machine interface and collision tracking.
