# Supplementary material for: Randomized resonant metamaterials for single-sensor identification of elastic vibrations
Source: Nat Commun. 2020 May 11;11:2353. doi: 10.1038/s41467-020-15950-1 (PMC7214442; doi:10.1038/s41467-020-15950-1)
Supplement: Supplementary file 3 — Description of Additional Supplementary Files [file 41467_2020_15950_MOESM3_ESM.pdf]

Description of Additional Supplementary Files

Supplementary Movie 1. The trajectory tracking process with 8 probes.

Supplementary Movie 2. The trajectory tracking process with 12 probes.
